# Control of bovine tuberculosis in a farmed red deer herd in England

**DOI:** 10.1136/vr.103930

**Published:** 2017-01-18

**Authors:** F. Busch, F. Bannerman, S. Liggett, F. Griffin, J. Clarke, K. P. Lyashchenko, S. Rhodes

**Affiliations:** 1 Animal and Plant Health Agency, Surrey, UK; 2 Scottish Agricultural College (SAC) Consulting: Veterinary Services, Inverness, UK; 3 University of Otago, Dunedin, New Zealand; 4 Enfer Scientific, Co Kildare, Ireland; 5 Chembio Diagnostic Systems, Inc., Medford, New York, USA

**Keywords:** Deer, Diagnostics, Mycobacterium bovis, Serology

## Abstract

This report describes how *Mycobacterium bovis* infection was controlled and eventually eradicated in a farmed red deer herd in the north of England, following sustained tuberculin skin testing supplemented with serological (antibody) tests over a period of approximately two years. By taking advantage of the anamnestic antibody response produced by the skin test to detect skin test-negative, antibody-positive infected individuals, a total of 35 additional animals were identified, including 2 with gross visible lesions typical of bovine tuberculosis (BTB). Without detection and removal, these animals would have posed a continued risk of BTB persistence within the herd and potentially contributed to the spread of infection from deer into wildlife and surrounding cattle farms in an area of low BTB incidence. This case supports the use of ancillary diagnostic serological tests to speed up the resolution of incidents of BTB caused by *M bovis* in captive deer herds.

There is no routine statutory bovine tuberculosis (BTB) testing programme for deer herds in Great Britain (GB); in England, the Tuberculosis (Deer and Camelid) (England) Order 2014 provides the statutory powers to require (skin) testing of deer herds infected with *Mycobacterium bovis* to be undertaken at the owner's expense in order to ascertain freedom from disease. In Scotland (officially BTB-free since September 2009) and Wales, statutory (skin) testing of deer in confirmed infected deer herds is funded by the Scottish and Welsh governments respectively under the Tuberculosis in Specified Animals (Scotland) Order 2015 and the Tuberculosis (Wales) Order 2011.

## Identification of infection in the deer herd

In January 2012, the Animal and Plant Health Agency (APHA) received a notification of TB lesions in a single deer that had been slaughtered on a farm in Yorkshire. The farm of origin was placed under movement restrictions and, following the isolation of *M bovis* in laboratory cultures, APHA undertook testing of the deer herd using the single comparative cervical tuberculin skin test as per existing guidelines. A total of 953 deer were tested, in which 26 reactors and 29 inconclusive reactors (IRs) were identified using the severe interpretation of the Single Intradermal Comparative Tuberculin Skin Test (SICCT) following infection confirmation. Skin reactors were found in hinds and stags, and higher numbers of reactors were identified in calves, suggesting widespread infection within the herd. All test reactors and IRs were slaughtered and postmortem examinations (PMEs) were conducted by the APHA veterinary staff at APHA laboratory facilities and in a dedicated local deer abattoir. Visible lesions (VLs) were found in BTB reactors from all deer groups, including the IRs. PME tissue samples were submitted to APHA Weybridge for mycobacterial culture and 10 further individuals were found to be *M bovis*-culture positive. Molecular typing (spoligotyping and variable nucleotide tandem repeat) of these *M bovis* isolates identified two genotypes: 9:f (thought to have been introduced to the Yorkshire herd after approximately 75 deer were moved from a Dorset premises in 2005) and genotype 21 (for which source tracings yielded no positive results).

## Wildlife investigations in the deer farm

Badger surveys were undertaken by the APHA National Wildlife Management Centre in May 2013 and November 2014 to ascertain the level of badger activity on the premises and to carry out a qualitative assessment of the risk of spillover of infection from the deer herd to local badgers and potentially to local cattle herds (e.g. including sett use, number of latrines, badger runs, etc, around the premises). The surveys concluded that there was a moderate to low level of badger activity around the deer housing and pasture on the affected farm and made a number of recommendations to reduce the risk of infection spillover onto the local badger population.

## BTB testing of co-grazing cattle in the deer farm

A number of deer from the herd had been co-grazed on the farm with a beef suckler herd consisting of 238 cattle under a contractual arrangement with Natural England. These cattle were also restricted, and with agreement from Natural England were fully separated from the deer by September 2013. The cattle herd had been subjected to TB testing every four years and remained officially BTB-free since it was established in 1990, with the last routine herd test conducted in 2010. A programme of skin testing was conducted in the cattle once separated from the deer to ensure freedom from infection. Between September 2012 and June 2015, a total of 1366 cattle skin tests were performed over eight rounds of testing as part of an enhanced cattle surveillance programme with no reactors identified. In June 2014, restrictions were lifted from the cattle business but regular check tests (every three to six months) were continued, with clear tests. Due to the severity of infection in the deer herd, repeat targeted testing of cattle within a 3-km radius was also conducted, during which time these contiguous herds were required to test any cattle moved to other farms. Only one test reactor (with no VLs (NVLs) and negative culture results) was found during the first round of targeted BTB surveillance in these contiguous cattle herds. Following completion of the herd tests with negative results, the radial testing zone was finally lifted in July 2015.

## BTB testing in cervids: options and usefulness

While the SICCT test is accepted to have high levels of sensitivity and specificity in cattle—80 per cent and 99.98 per cent respectively at standard interpretation (Karolemeas and others 2012, Goodchild and others 2015), there is less data on the performance of the SICCT in cervids. The sensitivity of the SICCT in 60 experimentally infected farmed deer in New Zealand was reported as 91 per cent (using a cut-off of 2-mm increase in the bovine tuberculin (purified protein derivative, bovine (PPDB)) response that was greater than or equal to the avian tuberculin (purified protein derivative, avian (PPDA) reaction) and an associated specificity (1157 BTB-free deer) of 99 per cent (Corrin and others 1993). In Spain, the SICCT in naturally infected wild fallow deer was estimated to have a sensitivity of 80 per cent (17 positive of 21 confirmed infected deer using a PPDB response increase of ≥2 mm and also ≥1 mm larger than the response to PPDA) (Jaroso and others 2010). Finally in the USA, the SICCT (following United States Department of Agriculture Veterinary Services Guidelines) provided 97 per cent sensitivity, but lower (81 per cent) specificity in white-tailed deer (Palmer and others 2001). There is no published sensitivity or specificity data for the SICCT for GB cervids.

The uncertainty over the effectiveness of the SICCT in cervids has led to studies of supplementary blood tests that, when used together with a tuberculin skin test, can increase the sensitivity and/or specificity of BTB infection detection: The Dual Path Platform (DPP) VetTB rapid immunochromatographic antibody test (Chembio Diagnostic Systems, Medford, New York, USA) has a published sensitivity and specificity in US white-tailed deer of 65 per cent and 98 per cent, respectively (Lyashchenko and others 2013), and in Spain the DPP VetTB test used in parallel with the comparative skin test (interpretation as in Jaroso and others 2010) was found to increase the sensitivity of detection of confirmed infected individuals from 76 per cent (skin test alone) to 97 per cent (skin test-positive plus DPP VetTB-positive combined) (Boadella and others 2012). A more dramatic example of an ineffective skin test being compensated by serology was described in a herd of farmed elk and fallow deer in Nebraska (Waters and others 2011). In this case, the serology tests applied (STAT-PAK, DPP VetTB and multi-antigen print assay (MAPIA)) provided a high level of sensitivity of infection detection (79–97 per cent), while the number of skin reactors was minimal. Finally, in New Zealand an antibody ELISA test, the ETB (Griffin and others 1991, 2004, 2005) was included into the successful TB-Free New Zealand Programme of the Animal Health Board Deer TB Tester Guidelines in 2009 (http://www.tbfree.org.nz/Portals/0/Deer%20TB%20Tester%20Guidelines%20Issue%201.pdf), which resulted in the reduction of deer-infected herds from 237 to just 4 in 10 years. In this case, the ETB was used primarily as a serial test to add specificity to positive single intradermal skin test (SIT) reactor results—the SIT having a higher sensitivity but lower specificity relative to the SICCT.

The STAT-PAK lateral-flow antibody test (Lyashchenko and others 2008; Chembio Diagnostic Systems)—forerunner of the DPP VetTB test—had been in use at APHA for a number of years, with proven usefulness for the detection of BTB in species such as badgers (Chambers and others 2008) and South American camelids (Dean and others 2009, Rhodes and others 2012). A preliminary study of the STAT-PAK test in wild deer at APHA suggested high levels of sensitivity (86 per cent) for this test (Gowtage-Sequeira and others 2009). However, the STAT-PAK test had not until this point been applied as an ancillary test to assist in the active case management of a TB breakdown in captive deer in GB.

## Materials and methods

### Serum samples

From April 2014, all serum samples were collected 10–30 days post SICCT to take advantage of the anamnestic antibody boost (Harrington and others 2008). Serum samples (474) from deer shown in Table 1 were allowed to clot in the collecting tube and were then centrifuged (800*g*, 40 minutes at room temperature (RT)) in order to separate the serum fraction. Serum was decanted into a clean tube and frozen at −20°C until required for antibody testing.

**TABLE 1:** Summary of deer bovine tuberculosis tuberculin skin tests and serology tests

Date of commencement of the test	SICCT (std/sev)*	Number tested	R/IRs	Serology tests†	Seropositive
January 2012	Severe	953	26/29	ND	ND
January 2013	Severe	877	14/12	ND	ND
June 2013	ND	ND		42^DPP/ETB/En/SP^	9
September 2013	ND	ND		21^En^	1 (IR)
December 2013	Severe	310	16/16	ND	ND
April 2014	Severe	268	2/36	125^DPP/En/ETB^	15
December 2014	Severe	283	8/44	125^En/ETB^	11
March 2015	Severe	330	10/33	73^ETB^	0
	Standard		0/10		
September 2015	Standard	378	0/10	88^DPP/ETB^	0

Numbers of tuberculin skin tests (SICCT*) and serology tests† and test-positives (R/IR/seropositive) in the deer herd between 2012 and 2015

The STAT-PAK test is excluded from April 2014 serology test data in the table due to the inconsistency of this test with the other three tests applied, possibly resulting from a cross-reactivity of the STAT-PAK test with Johne's infection in this herd, as stated in the text

*std/sev (standard or severe) refers to the interpretation of the skin test applied

†Serology tests from April 2014 included all skin reactors (R) and inconclusive reactors (IRs)

DPP, Dual Path Platform VetTB lateral flow; En, Enferplex ELISA; ETB, New Zealand ELISA; ND, not done; SP, STAT-PAK lateral flow

### SICCT for cervids

The SICCT was carried out according to Defra and APHA guidelines. Briefly, 0.1 ml of tuberculins (PPDB and PPDA, Lelystad, Prionics, Switzerland) were injected intradermally into the cervical region and the skin induration of each injection site measured with callipers at time zero and 72 hours later. The *standard interpretation* of the test is applied in herds with no recent BTB history, herds with no evidence of infection at PME of recently disclosed reactors and for IRs. A positive standard test requires that PPDB>PPDA by greater than 2 mm. A standard IR is described as reactions where PPDB>PPDA by 2 mm or less. The *severe interpretation* of the test is applied for herds confirmed with *M bovis* infection by microbiological culture. A positive severe test requires that PPDB≥PPDA. A severe IR is described as reaction to both PPDB and PPDA where the PPDB response is within 0.5–2 mm less than the PPDA response. The SICCT for cattle was applied using a standard interpretation, where PPDB>PPDA by greater than 4 mm.

### Serological tests

#### The STAT-PAK antibody test

The STAT-PAK antibody test (Chembio Diagnostic Systems) (Lyashchenko and others 2008, 2013) uses selected mycobacterial antigens (MPB83, ESAT6 and CFP10) immobilised on a nitrocellulose strip and a blue latex signal detection system for rapid detection of antibodies. The test was carried out according to the manufacturer's instructions. One test (cassette) was used per animal. Tests were carried out at RT. 30 μl serum was dispensed into the sample well of the cassette and allowed to soak into the wick. Three drops of sample buffer (included in the kit) were then added to the sample well. The test was incubated at RT for 20 minutes and the results noted. For a valid test, a complete blue band must appear across the control line site. For a positive test, a complete blue band must also appear at the test line site.

#### DPP VetTB rapid antibody test

The DPP VetTB test (Chembio Diagnostic Systems) (Lyashchenko and others 2013) consists of two nitrocellulose strips inside a cassette that allows independent delivery of test sample and antibody-detecting reagent to two antigen test lines (MPB83 and ESAT6/CFP10). One cassette was used per animal. Tests were carried out at RT. 5 μl serum was dispensed into the sample well followed by three drops of sample buffer. After five minutes, a further four drops of sample buffer were added to the buffer-only well. Cassettes were incubated for a further 20 minutes. For a valid test, a complete band must appear across the control line site. For a positive test, a complete band must also appear at one of the two test line sites.

### Enferplex ELISA

The Enferplex ELISA (Enfer Scientific, Kildare, Ireland) was carried out at the Enfer Scientific Laboratories as previously described (Whelan and others 2008) with some modifications for the detection of BTB in non-bovines. Serum samples were diluted 1:500 and 50 μl of the diluted sample added per test well. The microtitre plates were then incubated at RT with agitation for one hour. The plates were washed and the detection antibody (Protein G coupled to horse radish peroxidase (HRP)) was added (50 μl/well) and the plates incubated at RT for 30 minutes with agitation. The plates were washed as above and 50 μl of substrate added per well. Signals as relative light units were captured and data were extracted and analysed as previously described. Positivity was calculated based upon the reactivity to each of the seven specific antigens (PPDB, MPB83, MPB70, MPB70 peptide, Rv3616c, ESAT6 and CFP10) in the multiplex ELISA, with two spots positive resulting in a positive test readout.

### ETB ELISA

The ETB ELISA was carried out at the University of Otago as previously described (Griffin and others 2004) with modification (Griffin and others 2005). Briefly, ELISA plates were coated with the antigens PPDB, PPDA and MPB70. Serum samples were diluted 1:100 and added to the plates for one hour at 37°C, plates were washed and mouse-anti-cervid IgG1 was then added for a further 30 minutes at 37°C. Plates were again washed and results visualised using goat-anti-mouse-IgG-HRP followed by an o-phenylenediamine substrate system. Antibody titres were used to calculate the ETB result, with response to PPDB minus the response to PPDA≥20 indicating a positive result. An additional positive to MPB70 served to support the PPD result (increased confidence of a true positive).

## Results of BTB tests in the deer herd

Table 1 shows a summary of the various rounds of skin and serology tests carried out in this deer herd between January 2012 and September 2015. The SICCT was initially carried out at severe interpretation following the isolation of *M bovis* in the herd. In the last two herd tests, this was switched to the standard interpretation when there remained no further evidence of persistent infection in skin reactors or IRs. Serology testing began in June 2013 and continued, in one form or other, up to and including the final herd test that led to the lifting of restrictions on January 26, 2016. Serology was applied to all animals regardless of skin test status wherever possible. Thus, serology was applied in both serial (with skin test reactivity) and parallel (in the absence of skin reactivity) capacity. Positive skin reactors were in any case removed on the basis of their skin test status (i.e. severe interpretation reactors during the breakdown and standard interpretation reactors towards the end of the breakdown when no further evidence of infection was apparent despite extensive testing). The infection status of severe IRs however was more contentious from the outset due to the very stringent skin test criteria, and their removal could be dependent upon serological test outcome. Serology testing towards the end of the breakdown also provided confidence that infection had been cleared from the herd, allowing a change of SICCT interpretation from severe to standard.

In June 2013, the initial application of the STAT-PAK test (at APHA) on 42 yearling hinds from an epidemiological group of ∼150 animals in which several individuals had been identified with culture-positive results yielded seven (SICCT-negative) STAT-PAK-positives (all NVL—no culture performed). Later that year the owner organised for 21 (SICCT-negative) stags to be privately tested using the Enferplex test via Synergy Farm Health, Dorset, with one animal returning an ‘inconclusive’ result. The owner was advised to keep this animal isolated, but in December 2013 the animal was identified as a severe SICCT reactor and slaughtered. This animal had NVL at PME and was negative on culture.

In March 2014, APHA gave permission for the application of further serological testing in this deer herd, alongside the STAT-PAK and on a voluntary basis, using antibody tests/assays that had potential for BTB detection in cervids:

- The DPP VetTB test—supplied by Chembio Diagnostics Systems (‘DPP VetTB Assay for Cervids’) for the purpose of this trial (the test was not yet available at APHA) —test performed at APHA-Weybridge.
- The ETB ELISA, which had formed part of the successful New Zealand BTB control programme in deer since 2003—test performed at the University of Otago, New Zealand, via collaboration with Scottish Agricultural College (SAC) Consulting.
- The Enferplex ELISA, which had been used once previously in this herd and had published credentials for cattle (Whelan and others 2008, 2010, Casal and others 2014), camelids (Rhodes and others 2012) and goats (Shuralev and others 2012)—this test was carried out at the Enfer Scientific Laboratory in Ireland.
- The STAT-PAK test was carried out at APHA Starcross.

All test data were compiled independently of the testing laboratories.

In April 2014, 125 yearlings and hinds that were destined for the breeding herd were sampled for serology testing. In addition, serum samples from the previous 42 yearling hinds (June 2013) were tested retrospectively with the DPP VetTB, ETB and Enferplex tests. The STAT-PAK test returned 83 positive results, while the DPP VetTB, ETB and Enferplex tests collectively identified a much smaller group of 15 seropositive animals—the authors therefore used these 15 seropositive individuals to compare test performance (the excessive number of STAT-PAK-positive-only deer appeared to indicate some other cross-reactivity with this particular test—see later text).

Table 2 shows the level of agreement between the serology tests in this small group of 15 test-positives from the 125 yearlings and hinds antibody tested in April 2014, and also the discrepancies that can exist between tests that share common antigens, as these tests do. These discrepancies may be attributed to the differences in the test platforms, methods and the sample sizes required for each.

**TABLE 2:** Comparative summary of seropositive deer test results (April 2014)

Animal	STAT-PAK	DPP VetTB	ETB	Enferplex
1	Positive	Positive	Positive	Positive
2	Positive	Positive	Positive	Positive
3	Positive	Positive	Positive	Positive
4	Positive	Positive	Positive	Positive
5	Positive	Negative	Positive	Positive
6	Positive	Positive	Negative	Positive
7	Positive	Negative	Positive	Positive
8	Positive	Positive	Negative	Negative
9	Positive	Positive	Negative	Negative
10	Positive	Negative	Negative	Positive
11	Positive	Negative	Negative	Positive
12	Negative	Positive	Negative	Negative
13	Negative	Negative	Positive	Negative
14	Negative	Negative	Negative	Positive
15	Negative	Negative	Positive	Negative

The comparative test-positivity in the 15 seropositives from a cohort of 125 deer tested using the four different serology tests in April 2014 is shown. Only those individuals with a positive test outcome in either of the DPP VetTB, ETB or Enferplex tests are shown, together with the corresponding STAT-PAK result for the individual

This table excludes those (73) individuals positive only to the STAT-PAK test due to the inconsistency of this test with the other three tests applied, possibly resulting from a cross-reactivity of the STAT-PAK test with Johne's infection in this herd, as stated in the text

DPP VetTB, Dual Path Platform VetTB; ETB, New Zealand ELISA

Of the 15 seropositive deer, two (animals 4 (severe skin reactor) and 7 (severe IR)) had VL on PME and were *M bovis* culture-positive (spoligotype 9:f), a further three seropositive severe IRs were NVL, culture-negative (animals 10, 11 and 15). There were a further two SICCT reactors identified from the group of 125 deer; one of these was STAT-PAK-positive only and VL (but culture-negative), while the other was antibody test-negative (all tests), was NVL and culture-negative. Of the 42 yearling hinds (June 2013 samples) retrospectively tested with DPP VetTB, ETB and Enferplex tests, a further two animals were identified (both STAT-PAK-negative); one DPP VetTB-positive and one ETB-positive: both animals were NVL.

Supplementary antibody testing thus showed that the infection had not been eliminated from the deer herd. However, antibody test-positivity (even those positive to all four tests, thereby increasing the confidence of testing efficacy) did not automatically translate into confirmation of infection by the presence of obvious BTB lesions or a positive *M bovis* culture. It should be remembered that antibody tests are a measure of host response to infection, not a measure of the presence of pathology, which takes time to develop—the time from infection being different for each individual.

A further SICCT of the deer breeding herd in December 2014 identified 8 reactors and 44 IRs. Approved serology was carried out on 125 animals, including all reactors and IRs—these were tested privately using the Enferplex (via Synergy Farm Health, Dorset) and the ETB test (via SAC Consulting). APHA was not approached for the STAT-PAK test following previous potential issues of cross-reactivity, nor was the DPP VetTB test yet available as a routine test at APHA. Of the 125 deer tested, 1 was ETB-positive (NVL and culture-negative), and a further 10 deer were Enferplex-positive. All eight skin reactors were NVL and serologically negative. All 44 IRs were ETB-negative. 3 of the 44 IRs (plus 7 skin-negatives) were Enferplex-positive. All 10 Enferplex-positive animals were removed (all NVL and culture-negative).

The owner continued with serological testing using the ETB test (via SAC Consulting). Between March 2015 and May 2015, the breeding herd underwent a further SICCT for which both the standard and severe interpretations of the SICCT were considered since VL or culture-positive animals had not been found in this herd since mid-2014 despite extensive testing. This round of SICCT testing identified 10 IRs at standard interpretation, and 10 reactors and 33 IRs at severe interpretation. Approved serology (ETB) was carried out on 73 animals, including all IRs and severe reactors from this group, with negative results and all remained on-farm.

In the absence of VL and culture-positive reactors, the last SICCT was reinterpreted as standard—with no reactors. Thus, the next herd SICCT test (September–December 2015) was carried out at standard interpretation, identifying only 10 IRs. A total of 88 deer were serologically tested with ETB (via SAC Consulting) and DPP VetTB (now available at APHA) tests, including all IRs plus individuals with skin responses to PPDA from the previous SICCT, and a group of close contacts with no skin reactions. All were seronegative. Movement restrictions were therefore finally lifted from this deer herd on January 26, 2016.

## Discussion

The concern over the effectiveness of the SICCT alone, and the fact that any BTB testing in deer in England is carried out at the owner's expense complicated the management of this BTB incident. There were limited options in place at APHA, and other serology tests were becoming available from various sources. This resulted in a combination of serological tests being applied at various time points, depending upon test availability and the confidence of the owner in the tests themselves. Importantly, however, from April 2014, (i) all supplementary serological tests were applied within 10–30 days of the SICCT to boost the sensitivity of the serology test(s) applied and (ii) all deer on the farm eventually were antibody tested. This report represents the first case of a deer BTB breakdown where there has been a continual application of supplementary serology with the skin test, up to and including the clearing test which returned this herd to BTB-free status.

The benefit of supplementary serology in this deer herd was evidenced by the identification of a significant number of seropositive individuals—many of which were positive to more than one antibody test—and most of which (34/35) were not skin reactors. Nor did severe IR status appear to correlate with seropositivity since many were seronegative; however, one of the four seropositive severe IRs in April 2014 was VL and culture-positive (none of the seronegative severe IRs were VL). Thus, the application of supplementary serology may bring a level of confidence to the removal of infected animals where there is doubt regarding skin test status.

The relative sensitivity of the serological tests cannot be determined from the data obtained from this herd study as test-positives were few and many of these were NVL and culture-negative. But the ETB, Enferplex and DPP VetTB tests all appeared to provide an improvement in specificity compared with the STAT-PAK test (now replaced by DPP VetTB), which the authors suspected to be sensitive to *Mycobacterium avium* subspecies *paratuberculosis* (MAP) infection in this deer herd. Buddle and others (2010) showed that New Zealand farmed red deer infected with MAP (the agent of Johne's) could generate a positive STAT-PAK result, and a reaction to MPB83 (shown using MAPIA), but that such MAP infection-driven false-positive results were not obtained using the DPP VetTB test. In agreement with these findings, Lyashchenko and others (2013) showed that white-tailed deer in the USA experimentally inoculated with MAP did not produce positive DPP VetTB reactions. The New Zealand ETB ELISA also has published data to support the mitigation of this test against false-positive readouts caused by MAP, involving a modified readout when MAP interference/herd infection is suspected (Stringer and others 2011). Relevant to this current study, a case of Johne's disease had been diagnosed in the cattle herd co-located on the deer farm some years before. Following discussions with the owner, the authors reasoned that MAP infection could have been responsible for the relatively high number of STAT-PAK-positive test results in the deer herd compared with the other three antibody tests.

Notwithstanding the potential MAP interference, the otherwise high level of agreement that the authors found between serology tests in this study is similar to the concordance between antibody assays reported by Waters and others (2011) and further strengthens the case for supplementary serology in cervid BTB testing.

The questions raised by this case, regarding the availability, cost and validation of BTB testing regimes in infected deer herds, were raised as part of a Defra public consultation in 2015. The government's response to this consultation will be key if we are to engage the deer industry and individual owners in BTB herd health (21 separate deer BTB incidents in GB in 2014, https://www.gov.uk/government/uploads/system/uploads/attachment_data/file/457332/GB-surveillance-report14.pdf).

This herd case study suggests that we should consider the addition of supplementary serological testing as part of future BTB testing regimes for farmed deer herds with culture-confirmed *M bovis* infection. Serological testing to remove infected individuals not reactive to the SICCT (parallel testing) could enhance the sensitivity of infection detection during the course of a confirmed breakdown, while serial use towards the end of a breakdown could provide confidence for the true infection status of severe interpretation SICCT reactors and IRs, thus allowing for a return to a standard SICCT interpretation, and an exit strategy for deer herds to test out of restriction. Further test evaluation is recommended, incorporating both BTB-infected and BTB-free deer herds in order to provide the necessary data for test sensitivity and specificity respectively under GB conditions and to inform future discussion on the best use of such ancillary testing.
